# Tea consumption and the risk of biliary tract cancer: a systematic review and dose–response meta-analysis of observational studies

**DOI:** 10.18632/oncotarget.16963

**Published:** 2017-04-08

**Authors:** Jianping Xiong, Jianzhen Lin, Anqiang Wang, Yaqin Wang, Ying Zheng, Xinting Sang, Yiyao Xu, Xin Lu, Haitao Zhao

**Affiliations:** ^1^ Department of Liver Surgery, Peking Union Medical College Hospital, Chinese Academy of Medical Sciences and Peking Union Medical College, Beijing, China; ^2^ Department of Interventional Radiology, The First Affiliated Hospital of China Medical University, Shenyang, China; ^3^ State Key Laboratory of Quality Research in Chinese Medicine, Institute of Chinese Medical Science, University of Macau, Macau SAR, China

**Keywords:** tea, biliary tract cancer, cholangiocarcinoma, bile duct cancer

## Abstract

Recent studies have shown that tea consumption is associated with the reduced incidence of some types of cancer, possibly including biliary tract cancer. However, the epidemiological evidences for the association with risk of biliary tract cancer are contradictory. Thus, we performed meta-analysis of published observational studies to assess the association between tea consumption and risk of biliary tract cancer. Relevant studies were identified by searching PubMed, EMBASE, and ISI Web of Science published before October 2016. The Newcastle–Ottawa Scale was used to evaluate the quality of included studies, and publication bias was evaluated using funnel plots, and Begg's and Egger's tests. This meta-analysis includes eight studies comprising 18 independent reports. The incidence of biliary tract cancer reduced about 34% (significantly) for tea intake group in comparison with never intake group (summary odds ratio [OR] = 0.66; 95% confidence interval [CI] = 0.48–0.85). Additionally, an inverse relationship between tea intake and risk of biliary tract cancer was statistically significant in women (OR = 0.65; 95 % CI = 0.47–0.83), but not in men (OR = 0.86; 95% CI = 0.58–1.13). Dose– response analysis indicated that the risk of biliary tract cancer decreased by 4% with each additional cup of tea one day (relative risk [RR] = 0.96, 95% CI = 0.93–0.98, *p* = 0.001). In summary, tea intake is associated with decreased risk of biliary tract cancer, especially for women.

## INTRODUCTION

Biliary tract cancer, including gallbladder cancer (GC), extra hepatic bile ducts cancer (EXHBDC) and ampulla of Vater cancer (VOA) [1], is relatively rare, highly malignant, and has a high mortality rate. The overall 1-, 3- and 5-year relative survival rates are reportedly 25.0%, 9.7% and 6.8%, respectively, and have barely changed in the past few decades [2]. Although the incidence of biliary tract cancer is relatively low worldwide, the incidence is relatively high in some countries and regions of Latin America and Asia, for example, Japan (4/100 000 women), Chile (16.6/100 000 women), India (8.5/100 000 women), Korea (5.6/100 000 women), and Shanghai, China (5.2/100 000 women) [3]. About 7500 individuals are diagnosed with biliary tract cancer annually in the USA [4]. However, the causes of biliary tract cancer are still not well understood. Only a few risk factors have been identified, including gallstone disease, primary sclerosing cholangitis, and biliary tract infection [5–9]. Some studies have reported other possible risk factors for biliary tract cancer, including parity or age at first birth [10], diabetes [11], obesity [12], smoking [13], medical conditions, and family history of cancer [14]. However, the relationship between diet, especially tea, and biliary tract cancer is not yet well understood.

Tea is one of the most popular beverages worldwide, especially in Asia. Meantime, it is becoming more and more popular in the West. It is a drink for thousands of years with now growing interests in its additional benefits in health. For example, Studies have indicated that tea intake was associated with the reduced incidence of cardiovascular disease and increased bone density [15, 16]. In addition, tea is also associated with reduced risk of many types of cancer, including oral, bladder, esophageal, lung, breast, stomach, and liver cancer [17–27]. Although many observational studies have reported the relationship between tea and biliary tract cancer [28–35], no published studies could confirm the relationship. Therefore, we performed meta-analysis to present the association of them more comprehensive.

## RESULTS

### Study selection and study characteristics

**Figure 1:**
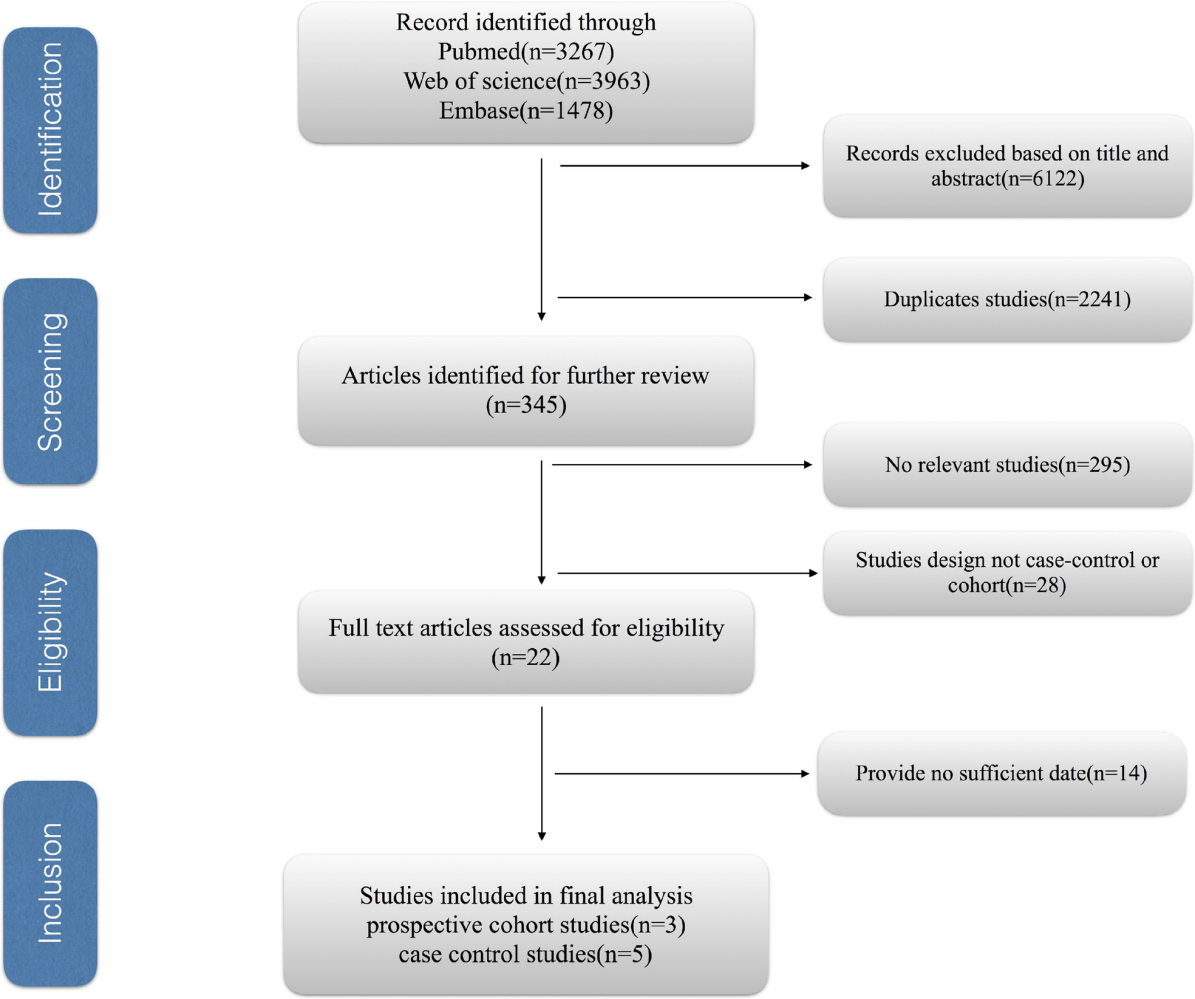
The process of selecting studies for the meta-analysis

Figure 1 shows the process of selecting studies for the meta-analysis. We obtained 8708 articles through the initial search, 2441 of which were duplicated. We further excluded 6122 studies based on title and abstract review. Finally, we identified eight eligible observational studies for our meta-analysis, including five case–control and three cohort studies.

The main characteristics of the included studies are listed in Supplementary Table 3. Two of them were performed in the USA, two in China, two in Japan, one in Poland, and one in Italy. There were a total of 7968 cases with biliary tract cancer in these studies, including 3802 with GC, 3808 with EXHBDC, and 358 with VOA. The duration of follow-up ranged from 2 to 13 years. The NOS scores for case control studies ranged from 6 to 8, with three high quality studies and two medium quality studies (Supplementary Table 1). All cohort studies were of high quality studies (Supplementary Table 2).

### Overall results

**Figure 2:**
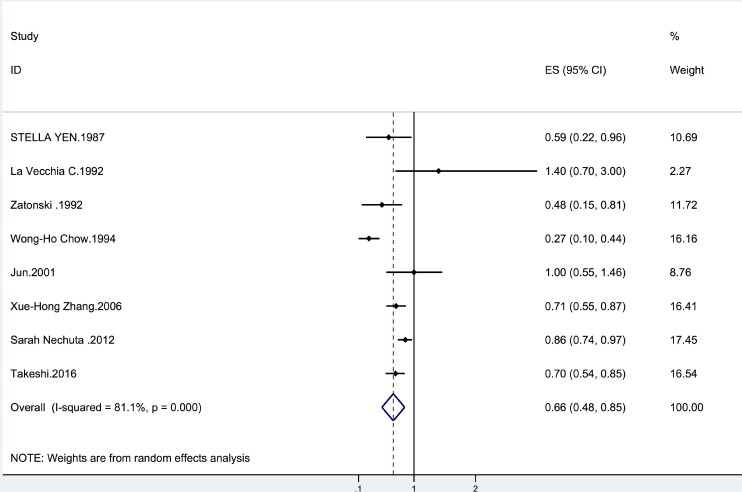
Forrest plot showing the relationship between tea and the risk of biliary tract cancer Points represent the risk estimate for each individual study. horizontal lines represent 95% confidence interval; diamonds represent the summary risk estimate with 95% confidence interval. CI, confidence interval. ES, effect size.

Overall, we found that tea intake was associated with a reduced incidence of biliary tract cancer (summary OR = 0.66, 95% CI = 0.48–0.85; *I*^2^ = 81.1%, *p* = 0.001) (Figure 2). The results were similar for tea consumption and GC risk (summary OR = 0.72, 95% CI = 0.56–0.88; *I*^2^ = 56.1%, *p* = 0.044) (Table 1). We also found that tea intake was associated with a significantly lower risk of EXHBDC (OR = 0.80, 95% CI = 0.71–0.89; *I*^2^ = 0.3%, *p* = 0.404) (Table 1). However, the inverse relationship between tea intake and the risk of VOA cancer was not statistically significant (OR = 0.78, 95% CI = 0.49–1.08; *I*^2^ = 59.8%, *p* = 0.083) (Table 1).

**Table 1:** Subgroup analyses for tea consumption on risk of biliary tract cancer

Subgroup	No. of studies	RR (95% CI)	*I*^2^value (%)	*P* value
**All studies**	8	0.66 (0.48, 0.85)	81.1	0.001
Subtype of cancer				
GC	6	0.72 (0.56, 0.88)	56.1	0.044
EXHBDC	5	0.80 (0.71, 0.89)	0.3	0.404
VOA	4	0.78 (0.49, 1.08)	59.8	0.083
Study design				
case-control	5	0.62 (0.44, 0.80)	55.8	0.009
cohort	3	0.84 (0.77, 0.90)	0.6	0.001
Gender				
male	2	0.86 (0.58, 1.13)	0	0.650
female	3	0.65 (0.47, 0.83)	86.7	0.001
male and female	5	0.72 (0.57, 0.87)	24.0	0.246
Geographic areas				
West	4	0.45 (0.24, 0.65)	36.2	0.152
East	4	0.81 (0.74, 0.88)	21.4	0.001
No. of case				
≥ 200	3	0.79 (0.70, 0.88)	48.9	0.081
< 200	5	0.68 (0.47, 0.88)	61.9	0.002
Publication time				
≥ 2000	4	0.81 (0.74, 0.88)	21.4	0.240
< 2000	4	0.45 (0.24,0.65)	36.2	0.152
Duration of fallow-up				
≥ 5	2	0.83 (0.76, 0.90)	11.6	0.340
< 5	6	0.65 (0.47, 0.83)	58.5	0.004
Study quality				
≥ 7	6	0.80 (0.73, 0.88)	25.7	0.207
< 7	2	0.55 (0.31, 0.80)	56.5	0.024
**Adjustment for confounders**				
cholelithiasis				
Yes	2	0.70 (0.54, 0.85)	0	0.367
No	6	0.71 (0.58, 0.85)	72.4	0.001
smoking				
Yes	4	0.62 (0.38, 0.87)	70.3	0.001
No	4	0.79 (0.70, 0.88)	37.2	0.111
Body Mass Index				
Yes	3	0.84 (0.77, 0.90)	0.6	0.412
No	5	0.62 (0.44, 0.80)	55.8	0.009
Eduction				
Yes	5	0.81 (0.73, 0.90)	30.3	0.158
No	3	0.54 (0.32, 0.75)	69.5	0.008

RR, relative risk; CI, confidence interval.

### Subgroup and sensitivity analyses

The results of subgroup analyses are shown in Table 1. When the analysis was stratified by sex, we found tea was associated with a significantly lower risk of biliary tract cancer in women (OR = 0.65, 95% CI = 0.47–0.83; I^2^ = 86.7%, *p* = 0.001) rather than in men (OR = 0.86, 95% CI = 0.58–1.13; I^2^ = 0.6%, *p* = 0.650) (Table 1). According to sensitivity analyses, by excluding studies that were ineligible for dose–response analysis, the relationship between tea intake and biliary tract cancer still remain stable (OR = 0.77, 95% CI = 0.67–0.88; I^2^ = 41.5%, *p* = 0.114) (Table 1).

### Dose–response meta-analysis

**Figure 3:**
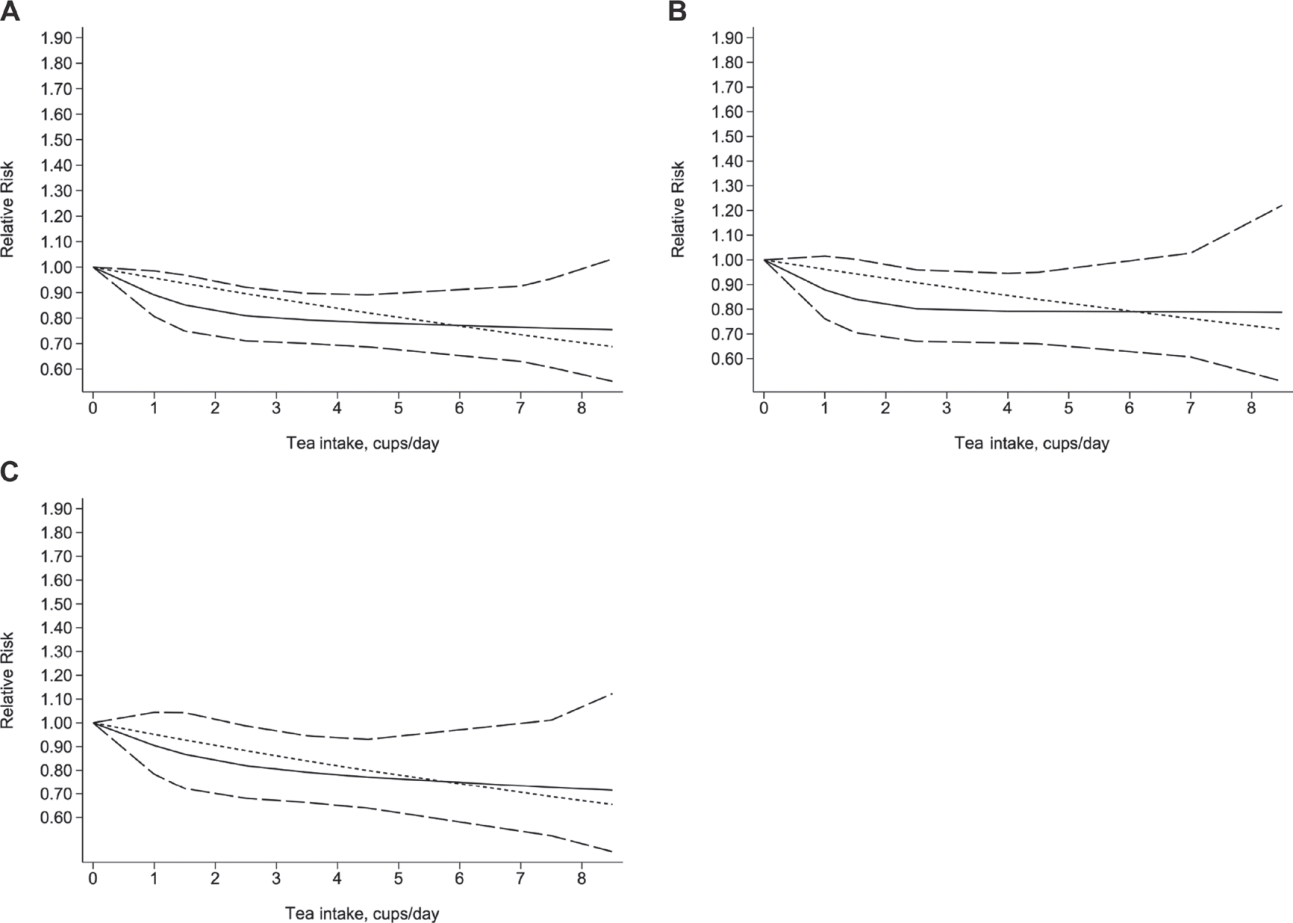
Dose-response relationship between tea intake and the risk of biliary tract cancer (**A**), gallbladder cancer (**B**), extra hepatic bile ducts cancer (**C**). The solid line and the long dash line represent the estimated relative risks and its 95% confidence interval. Short dash line represents the linear relationship.

Five studies (three case control and two cohort) with a total of 7011 patients with biliary tract cancer were eligible for the assessment of the dose–response relationship between tea intake and the risk of biliary tract cancer. When we used the restricted cubic splines model, we found that the test of a nonlinear relationship between tea intake and biliary tract cancer was rejected (p for nonlinearity = 0.1902). Therefore, we identified a linear relationship with a linear regression model (p for linearity = 0.0004). We found that the risk of biliary tract cancer decreased by 4% with each additional cup of tea one day (RR = 0.96, 95% CI = 0.93–0.98, p = 0.001) (Figure 3). In addition, when we stratified the dose–response analysis by subtype of cancer, we obtained similar result for GB cancer (p for nonlinearity = 0.1902; p for linearity = 0.0004) and EXHBDC (p for nonlinearity = 0.6394; p for linearity = 0.0064). Each additional cup/day of tea was associated with a 4% decreased risk of GB cancer (RR = 0.96, 95% CI = 0.93–0.99, *p* = 0.024) (Figure 3) and 5% EXHBDC (RR=0.95, 95% CI = 0.92–0.99, *p* = 0.006) (Figure 3), respectively. There were too few cases of VOA (*n* < 3) to perform a dose–response analysis.

### Publication bias

**Figure 4:**
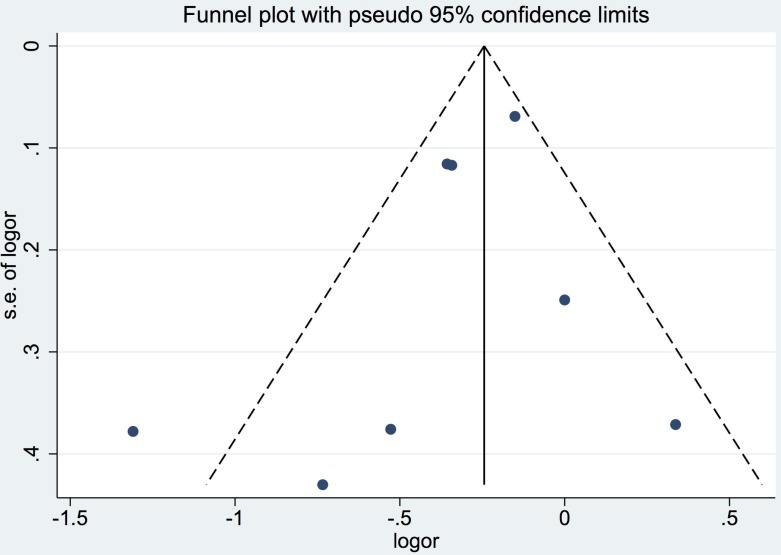
Funnel plot for studies included in the meta-analysis of the relationship between tea intake and biliary tract cancer risk LogOR: Log odds ratio. SE: standard error.

Although the number of studies included in the meta-analysis was less than ten, the funnel plots still did not reveal substantial asymmetry. Additionally, Begg's and Egger's tests did not identify substantial publication bias (*p* > 0.05) (Figure 4).

## DISCUSSION

As far as we know, this is the most comprehensive study to explore the relationship between tea and biliary tract cancer. We found that tea intake is associated with a 34% lower incidence of biliary tract cancer (OR = 0.66; 95% CI = 0.48–0.85). Subgroup analysis by type of cancer yielded similar results for risk of GB cancer (OR = 0.72; 95% CI = 0.56–0.88) and EXHBDC (OR = 0.80, 95% CI = 0.71–0.89; I^2^ = 0.3%, *p* = 0.404), respectively. However, we did not find a statistically significant inverse relationship between tea intake and the risk of VOA (OR = 0.78; 95% CI = 0.49–1.08). Additionally, we identified a statistically significant inverse relationship between tea intake and the risk of biliary tract cancer in women (OR = 0.65; 95% CI = 0.47–0.83), but not in men (OR = 0.86; 95% CI = 0.58–1.13).

In the dose-response analysis, we found a 4% decreased risk of biliary tract cancer (RR = 0.96, 95% CI = 0.93–0.98, *p* = 0.001) with each additional cup/day. Furthermore, when we stratified the dose–response analysis by type of cancer, we found the risks of GB cancer and EXHBDC decreased by 4% and 5%, respectively.

An important ingredient of tea is tea polyphenols, which are an important type of antioxidant. Tea polyphenols, also known as catechins, have four important types of component, namely (−)-epigallocatechin gallate (EGCG), (−)-epigallocatechin (EGC), (−)-epicatechin gallate (ECG), and (−)-epicatechin (EC) [36]. EGCG is the main tea catechins and also plays the most important role in inhibiting the formation of cancer [37]. Firstly, it can suppress the growth of cancer cells and induce their apoptosis [38]. Moreover, it can suppress receptor-dependent signaling pathways and angiogenesis, thus preventing tumorigenesis [39]. Secondly, a common characteristic of tumor cells is methylation of DNA, which plays an important role in epigenetic mechanisms for silencing various genes. EGCG contained in the tea polyphenols can inhibit this biochemical process, thus preventing or reversing related gene-silencing in cancer cells [40]. Thirdly, tea polyphenols can inhibit activities of enzyme, including DNA methyltransferase, dihydrofolate reductase, glucose-6-phosphate dehydrogenase, and glyceraldehyde-3-phosphate dehydrogenase, thus preventing cancer formation [41]. Although many studies have suggested that tea can prevent cancer formation and have elucidated the mechanism, the mechanism about tea and biliary tract cancer is still unclear.

Our study has several strengths. First, it is the first to explore the dose–response relationship between tea intake and the risk of biliary tract cancer. Second, we performed subgroup and sensitivity analyses to determine the factors affecting the risks. Third, most of the studies included in our meta-analysis were of high quality. All of these characteristics make the conclusions of our study more convincing.

However, there are still several limitations that must be taken into account. First, there are many type of teas, including green, black, and oolong tea [42]. However, we were unable to retrieve information about the type of teas, which might have influenced the virtual results. For example, researcher has reported that green tea reduces cancer risk more strongly than black tea [43]. Secondly, the heterogenicity among studies was obvious and acted as another potential limitation of this study. Third, although we did address a number of adjustment factors, we could not address all potential adjustment factors. People with biliary cancer may stop drinking caffeine-containing beverage and pay more attention on physical exercise, which might have influenced the impact on risk. Finally, our study comprised five case control and only three cohort studies. The former was prone to generate recall and selection biases.

In summary, we found that tea is associated with a 34% lower incidence of biliary tract cancer. Subgroup analysis showed that tea consumption is associated with decreased risk of GB and EXHBDC with 28% and 20%, respectively. However, more prospective studies and basic research are still urgently needed to further validate the association between tea and biliary cancer and the potential mechanisms.

## MATERIALS AND METHODS

### Data sources and search strategy

We searched published reports in the PubMed, EMBASE and Web of Science using the following keywords: (“tea OR beverages OR diet OR drinking OR risk factor”) and (“gallbladder cancer” OR “gallbladder carcinoma” OR “gallbladder tumor” OR “gallbladder neoplasms” OR “biliary tract cancer” OR “bile duct cancer” OR “cholangiocarcinoma”). We placed no restrictions on the language or date of publication.

### Eligibility criteria for study selection

The eligibility criteria were as follows: study design (case control or cohort); exposure factor tea and outcome biliary tract cancer, including cancers of the GB, EXHBD, and VOA; and odds ratio (OR)/risk ratio (RR) values and corresponding 95% confidence intervals (CIs) for different categories of tea consumption available or sufficient information provided to enable the calculation of these variables. If two studies reported the same data, we selected the study with the larger sample.

### Data abstraction and quality assessment

Two researchers independently extracted the required information from the selected reports in a standardized manner. We collected the following information from each article: year of publication, first author's name and country of origin, study design (case control or cohort), number of participants (cases, controls, or cohort size), duration of follow-up, types of cancer, sex of participants, sources of controls, comparison of exposure levels, potential adjusted confounding variables, OR/RR values and 95% CIs for different categories of tea consumption, and quality score. To assess the dose–response, we also collected the number of case and person-years for each category of tea consumption.

We used the Newcastle– Ottawa Scale (NOS) [44] to evaluate the quality of included studies. We assigned quality categories based on the scores of each study. The categories were the following: high quality (score 7–9), medium quality (score 4–6) and low quality (score less than 4) [45]. We resolved discrepancies by consensus.

### Statistical analyses

We assessed the relationship between tea consumption and biliary tract cancer using OR/RR values and the corresponding 95% CIs. When the results provided were for multiple groups of tea consumption with OR or RR values and corresponding 95% confidence intervals, we combined them to obtain a single OR/RR value and corresponding 95% CI [45]. We treated the hazard ratio as equivalent to the RR. When separate results were reported for men and women, we analyzed the findings for men and women as two different independent reports. Additionally, when results for subtypes of biliary tract cancers, such as GB cancer, EXHBDC, and VOA, were reported, we analyzed data for each subtype of cancer as an independent report.

To enable the meta-analysis of the dose–response, we extracted the number of cases and person-years and RRs with variance estimates for at least three quantitative exposure categories from each study. If the studies did not provide these data, we required sufficient information to calculate them. If the intake of tea was reported as amount per year or lifetime, we calculated the daily intake. If studies did not provide tea intake in terms of cups, we assumed 120 mL or 50 g as one cup [31, 35]. For dose–response analysis, we used the midpoint of tea intake in each category as the dose of tea consumed. If the highest category was open- ended, we set the midpoint of the category at 1.5 times the lower boundary; if the lowest category was open-ended, we set the lowest boundary at zero [46]. Additionally, we used restricted cubic splines with four knots at fixed percentiles (5%, 35%, 65%, and 95%) of the distribution to evaluate a potential linear relationship between tea consumption and the risk of biliary tract cancer. A *p* value for the curve linearity relationship was calculated by testing whether the coefficient of the second spline was zero [47]. Greenland and Orsini were the pioneers of this method [48, 49] [50], we use the method to test a simple quadratic term to the linear model and it was based on the results across categories of tea consumption. Many subsequent studies have described it in detail [51, 52].

We used I^2^ to assess heterogeneity between studies and defined low, medium, and high heterogeneity as 25%, 50%, and 75%, respectively [53]. If p was less than 0.1, we assumed definite heterogeneity. We used the fixed effect model when the heterogeneity was not substantial and the random effects model when there was a significant heterogeneity [54]. We evaluated publication bias by funnel plots, with funnel plot asymmetry indicating the presence of bias [55].

We also performed subgroup analyses by subtype of cancer, study design, sex, geographic area, number of cases, date of publication, duration of follow-up, study quality (NOS scores), and whether cholelithiasis, smoking, body mass index or education was adjusted for in the models. Sensitivity analyses were also performed by excluding studies not eligible for dose–response analysis.

All statistical analyses were performed using STATA version 12.0 (Stata).

## SUPPLEMENTARY TABLES





## Abbreviations

GC: gallbladder cancer
EXHBDC: extra hepatic bile ducts cancer
VOA: ampulla of Vater cancer
