# Supplementary material for: Tea consumption and the risk of biliary tract cancer: a systematic review and dose–response meta-analysis of observational studies
Source: Oncotarget. 2017 Apr 8;8(24):39649–57. doi: 10.18632/oncotarget.16963 (PMC5503640; doi:10.18632/oncotarget.16963)
Supplement: Supplementary file 1 [file oncotarget-08-39649-s001.pdf]

Tea consumption and the risk of biliary tract cancer: a systematic review and dose–response meta-analysis of observational studies

Supplementary Materials

Supplementary Table 1: Scores of the Newcastle-Ottawa scale for case control studies

Study/Years of Publication	Fully defined cases	Representative cases	Selection of controls	Definition of controls	Controlling the important factors or confounding factors	Determination of exposure	Same method of determination for cases and control	Non-response rate	Total score
La Vecchia C.1992	*	*		*	**		*		6
Zatonski .1992	*	*	*		**	*	*		7
Xue-Hong Zhang.2006	*	*		*	**	*	*	*	8
Wong-Ho Chow.1994	*	*	*		**		*	*	7
STELLA YEN.1987	*	*	*		**		*		6

Supplementary Table 2: Scores of the Newcastle-Ottawa scale for cohort studies

Study/Years of Publication	representativeness of exposed cohort	Selection of the non-exposed cohort	Determination of exposure	outcome not present at study start	Controlling the important factors or confounding factors.	Assessment of outcome	Follow-up long enough for outcome to occur	Integrity of follow up	Total score
Jun.2001	*	*	*	*	*	*		*	7
Sarah Nechuta .2012	*	*	*	*	**	*	*	*	9
Takeshi.2016	*	*	*	*	**	*		*	8

Supplementary Table 3: The main characteristics of the included studies. See Supplementary_Table_3
