# Supplementary material for: Tea consumption and the risk of biliary tract cancer: a systematic review and dose–response meta-analysis of observational studies
Source: Oncotarget. 2017 Apr 8;8(24):39649–57. doi: 10.18632/oncotarget.16963 (PMC5503640; doi:10.18632/oncotarget.16963)
Supplement: Supplementary file 2 [file oncotarget-08-39649-s002.docx]

| **tea and EHBDC** | | | | | | | | |  |  |  |
| --- | --- | --- | --- | --- | --- | --- | --- | --- | --- | --- | --- |
| Study/Years  of  Publication | Country | No. Case/pesson-years | Follow | Sources of Controls | Gender | Subtype of cancer | Subtype of study | Exposure | Adjusted Factors | Comparison of Exposure Level | Adjusted OR/RR (95% CI) |
| STELLA YEN.1987 | usa | 41/141  25/86  23/66 | 1975-1979 | Population | male and female | EHBDC | case-control | tea | sex and age in decades, | never vs 1-2cups/d  never vs 3-4cups/d  never vs >5cups/d | 0.64(0.31,1.31)  0.46(0.15,1.40)  0.79(0.19,3.26) |
| Wong-Ho Chow.1994 | usa | 34/97 | 1985-1989 | Population | male | EHBDC | case-control | tea | age, ethnic origin, and smoking status. | never vs ever | 0.6 (0.3-1.6) |
| Wong-Ho Chow.1994 | usa | 30/157 | 1985-1989 | Population | female | EHBDC | case-control | tea | age, ethnic origin, and smoking status. | never vs ever | 0.2(0.1,0.5) |
| Xue-Hong Zhang.2006 | china | 99/373 | 1997-2001 | Population | male | EHBDC | case-control | tea | age at interview, education, | never vs ever | 1.03(0.64,1.64 ） |
| Xue-Hong Zhang.2006 | china | 92/586 | 1997-2001 | Population | female | EHBDC | case-control | tea | age at interview, education, | never vs ever | 0.65( 0.37,1.14 ) |
| Sarah Nechuta .2012 | china | 1077/901030  1077/901030  1085/901030 | 1996–2009 | Population | female | EHBDC | cohort | tea | age, marital status, education, occupation, BMI, exercise, fruit and vegetable intake, meat intake, diabetes, and family history of digestive system cancer | ≥3cups/d VS never  1cups/d VS never  2-3cups/d VS never | 0.79 (0.63, 0.99) 0.95(0.76,1.18) 0.85 (0.68, 1.06) |
| Takeshi.2016 | japan | 68/504235  78/582998  79/582722 | 1990-1999 | Population | male and female | EHBDC | cohort | tea | age, study area, sex, body mass index, history of cholelithiasis, history of diabetes mellitus, history of chronic hepatitis or cirrhosis, history of smoking, drinking fre- quency, physical activity by metabolic equivalents ⁄ day score, total energy consumption, energy-adjusted consumption of fish, red meat, and veg- etable and fruit, and coffee. | > 6cups/d VS 0-1cups/d 1-3cups/d VS 0-1cups/d 3-6cups/d VS 0-1cups/d | 0.69（0.41,1.15）  0.83（0.53,1.31）0.79（0.50,1.26） |

| **tea and ampulla of vater** | | | | | | | | |  |  |  |
| --- | --- | --- | --- | --- | --- | --- | --- | --- | --- | --- | --- |
| Study/Years  of  Publication | Country | No. Case/Control or Cohort Size | Follow | Sources of Controls | Gender | Subtype of cancer | Subtype of study | Exposure | Adjusted Factors | Comparison of Exposure Level | Adjusted OR/RR (95% CI) |
| Wong-Ho Chow.1994 | usa | 15/97 | 1985-1989 | Population | male | AOV | case-control | tea | age, ethnic origin, and smoking status. | never vs ever | 1.8(0.6,5.6) |
| Wong-Ho Chow.1994 | usa | 26/157 | 1985-1989 | Population | female | AOV | case-control | tea | age, ethnic origin, and smoking status. | never vs ever | 0.4(0.1,0.9) |
| Xue-Hong Zhang.2006 | china | 68/959 | 1997-2001 | Population | male and female | AOV | case-control | tea | age at interview, education, | never vs ever | 1.06(0.68,1.69) |
| Sarah Nechuta .2012 | china | 247/901030 | 1996–2009 | Population | female | AOV | cohort | tea | age, marital status, education, occupation, BMI, exercise, fruit and vegetable intake, meat intake, diabetes, and family history of digestive system cancer | curren  VS  never | 0.86 (0.74,0.98) |

| **tea and gallbladder cancer** | | | | | | | | |  |  |  |
| --- | --- | --- | --- | --- | --- | --- | --- | --- | --- | --- | --- |
| Study/Years  of  Publication | Country | No. Case/person-years | Follow | Sources of Controls | Gender | Subtype of cancer | Subtype of study | Exposure | Adjusted Factors | Comparison of Exposure Level | Adjusted OR/RR (95% CI) |
| La Vecchia C.1992 | Italy | 41/- | 1983-1987 | Hospital | male and female | GC | case-control | tea | age, sex, area of residence, education, smoking, coffee consumption | ever vs never | 1.4(0.7,3.0) |
| Zatonski .1992 | Polish | 48/98  39/111 | 1985-1988 | Population | male and female | GC | case-control | tea | age,sex and education. | <6790 vs none  >6790 vs none | 0.67(0.29,1.55)  0.41(0.18,0.96) |
| Jun.2001 | japan | 77/277743  55/160785 | 1979-1981 | Population | male and female | GC | cohort | tea | city,gender,age,radiation exposure,smoking status,acohol drinking,BMI,education level and calendar time | 2-4/day vs 0-1cups/d  >5/day vs 0-1cups/d | 0.9(0.57,1.7)  1.2(0.66,2.2) |
| Xue-Hong Zhang.2006 | china | 99/10492 | 1997-2001 | Population | male | GC | case-control | tea | age at interview, education, | never vs ever | 0.82(0.52,1.30) |
| Xue-Hong Zhang.2006 | china | 267/10492 | 1997-2001 | Population | female | GC | case-control | tea | age at interview, education, | never vs ever | 0.56(0.38,0.83) |
| Sarah Nechuta .2012 | china | 1077/901030  1077/901030  1085/901030 | 1996-2009 | Population | female | GC | cohort | tea | age, marital status, education, occupation, BMI, exercise, fruit and vegetable intake, meat intake, diabetes, and family history of digestive system cancer | ≥3cups/d VS never  1cups/d VS never  2-3cups/d VS never | 0.79 (0.63, 0.99) 0.95(0.76,1.18) 0.85 (0.68,1.06) |
| Takeshi.2016 | japan | 54/504235  53/582998  71/582722 | 1990-1999 | Population | male and female | GC | cohort | tea | age, study area, sex, body mass index, history of cholelithiasis, history of diabetes mellitus, history of chronic hepatitis or cirrhosis, history of smoking, drinking fre- quency, physical activity by metabolic equivalents ⁄ day score, total energy consumption, energy-adjusted consumption of fish, red meat, and veg- etable and fruit, and coffee. | > 6cups/d VS 0-1cups/d 1-3cups/d VS 0-1cups/d 3-6cups/d VS 0-1cups/d | 0.57（0.32,1.01）  0.56（0.32,0.97）0.88（0.54,1.45） |
